# Hospital and Asylum Construction

**Published:** 1895-10-19

**Authors:** 


### HOSPITAL & ASYLUMN CONSTRUCTION.

### MIDDLESEX LUNATIC ASYLUM.—NEW WING.

The foundation-stone of a new wing to this "Hospital for the Insane" was laid on Saturday last by Mr. H. R. Williams, the chairman of the committee, in the absence of the Lord-Lieutenant of the county, the Earl of Strafford, who was unavoidably prevented from being present. Previous to the laying of the stone, Mr. Williams explained to the visitors that the new buildings, which are to accommodate 200 patients, are to be devoted to the care and education of idiots and imbeciles—for whom half a century ago no provision existed at all. It was owing to the representations made by a benevolent lady to the Rev. Dr. Reed, an energetic philanthropist in his day, that a home was first instituted at Highgate, afterwards developing into the Colchester Asylum. Dr. Reed also founded the Earlswood Asylum. The speaker, in his remarks, laid stress on the fact that love should be the guiding principle in dealing with idiots and lunatics, and that would be the line followed by the attendants in the new buildings.

The new buildings, the plans for which have been prepared by Mr. Rowland Plumbe, are connected with the asylum proper by a spacious subway. The administrative block, two stories in height, will be the central figure, with the main entrance, over which will be a tower containing water-tanks for 10,000 gallons. Here will be also accommodation for head male and female attendants, assistant medical officers, &c. The ward blocks are placed at the four angles of a square, with the administrative block in the middle of the south side, with hall, kitchen, mess-rooms, and stores running across the centre, the general stores and back entrances being on the north. The construction throughout will be fireproof, and ample escape staircases will be provided. The arrangements altogether are to be on a most complete and thorough scale.

By reason of these additions the entire remodelling of the asylum laundry has been necessitated; the old buildings are to be removed, and new wards for seventy female epileptics will take their place. The new laundry will be on the newest and most approved principles; the water to be used will be subjected to a softening and filtering process before being conveyed to the various storage tanks. The various heating and steam boilers, pumps, engineers' workshops, &c., will be collected into one block, with a chimney shaft 120 feet in height. The total cost of all the additions and alterations is estimated at £99,500.

